# Gut microbiota dysbiosis in adolescent depression: a comparative analysis with adult depression and healthy adolescent

**DOI:** 10.3389/fmicb.2026.1849097

**Published:** 2026-07-09

**Authors:** Yongchun Ma, Liyuan Luo, Huichao Li, YuXin Zhou, Shasha Xiang, Xiaohong Liu, Xuan Zhu

**Affiliations:** 1 Department of Sleep Medicine, Tongde Hospital of Zhejiang Province, Hangzhou, Zhejiang, China; 2 School of Food Science and Bioengineering, Zhejiang Gongshang University, Hangzhou, Zhejiang, China; 3 Weifang Elbe Biotechnology Co., Ltd., Weifang, China

**Keywords:** adolescent depression, gut microbiota' microbiota-gut-brain axis, full-length 16S rRNA, gut microbiota, age-conserved, adolescent-specific

## Abstract

**Introduction:**

Adolescent depression differs substantially from adult depression in neurodevelopmental and pathophysiological aspects, with gut microbiota dysbiosis recognized as a key regulator via the microbiota-gut-brain axis. Nevertheless, the age-specific microbial and metabolic signatures of adolescent depression remain poorly defined.

**Methods:**

125 participants including 33 adolescents with depression, 62 adults with depression, and 30 healthy adolescent controls were recruited. Full-length 16S rRNA gene sequencing was applied to profile gut microbial composition and functional metabolism.

**Results:**

Alpha diversity was markedly reduced in adolescents with depression relative to healthy adolescent, whereas no significant difference was observed between adolescent and adult depression. PCoA and age-adjusted PERMANOVA revealed distinct microbial community separation across the three groups, with disease status contributing substantially to community divergence. A significant Group × Age interaction indicated that depression may exerted age-dependent effects on gut microbiota. At the genus level, 19 differential taxa were identified between depressed and healthy adolescents, with *Akkermansia, Faecalibacterium, Prevotella*, and *Roseburia* depleted, while only *Eubacterium hallii* group was enriched. Four genera differed between adolescent and adult depression, and 3 genera exhibited obvious age-interaction patterns, suggesting that adolescent depression disrupts microbial developmental trajectories and induces an adult-like dysbiotic phenotype. A Random Forest diagnostic model based on differential bacterial biomarkers achieved achieved promising exploratory performance in distinguishing depressed adolescents from healthy controls (5-CV AUC = 0.966), but failed to differentiate adolescent from adult depression. PICRUSt2 prediction further revealed that gut microbiota metabolism alterations in depression are primarily driven by depressive status.

**Discussion:**

Our findings reveal both age-conserved and adolescent-specific microbial signatures, providing candidate microbial signatures and hypothesis-generating functional insights.

## 1 Introduction

Major depressive disorder (MDD) represents one of the most prevalent psychiatric conditions globally, with lifetime prevalence exceeding 15% (Friedrich, 2017). According to the most recent data released by the World Health Organization (WHO), the global prevalence of depression approximates 4.40%, affecting an estimated 322 million individuals worldwide, thereby constituting the leading cause of disability across all age groups (Friedrich, 2017). This condition has emerged as a critical public mental health priority requiring urgent intervention. In recent years, China has witnessed a concerning escalation in the prevalence of depressive symptoms among adolescents. A comprehensive investigation conducted in 2019 reported that the detection rate of depression among Chinese adolescents reached an alarming 24.30% (Tang et al., 2019), severely compromising the normative physical and psychological development of this demographic. The consequences of untreated adolescent depression are profound and potentially irreversible, encompassing academic withdrawal, substance abuse, violent behaviors, and tragically, suicide.

The affective symptomatology of depression demonstrates intimate pathophysiological connections with gastrointestinal manifestations. The gastrointestinal tract plays a pivotal role in cerebral activity and emotional regulation. Epidemiological investigations have documented substantially elevated prevalence rates of psychiatric comorbidities among individuals with gastrointestinal disorders, estimated between 60% and 85% (Simpson et al., 2020; Huang et al., 2021). Conversely, patients diagnosed with depression frequently present with concomitant gastrointestinal symptomatology. Hao et al. (2024) demonstrated that murine models exhibiting depression-like behaviors displayed pronounced colonic inflammation, compromised barrier integrity, and concurrent alterations in gut microbial composition. Over the past decade, accumulating evidence has established that intestinal microbiota serve as critical modulators in the bidirectional communication between the brain and gastrointestinal system, leading to the conceptualization of the “microbiota-gut-brain axis” (Hao et al., 2024).

To elucidate the causal relationship between gut microbiota composition and depression pathogenesis, Gao et al. (2023) conducted a comprehensive systematic review and meta-analysis of previously published investigations. Their findings indicated that while α-diversity indices remained unchanged in depressed patients, significant alterations in species composition were evident, specifically characterized by reduced *Firmicutes* and elevated *Bacteroidetes* at the phylum level (Gao et al., 2023). These observations contradicted the findings reported by Kelly et al. (2015). Furthermore, Hao et al. (2024) documented decreased intestinal microbial α-diversity in depression-like mice, accompanied by increased abundance of *Proteobacteria*. Cryan and Dinan (2012) proposed that potentially pathogenic members of *Proteobacteria* may influence cerebral function through toxin production and inflammatory pathway activation, ultimately precipitating depressive symptomatology. Gao et al. (2023) additionally identified reductions in *Eubacterium_ventriosum_group, Faecalibacterium*, and five other genera, alongside elevations in *Eggerthella, Enterococcus, Escherichia, Flavonifractor, Lachnoclostridium, Streptococcus*, and two additional genera at the genus level. Conversely, Stephanie et al. reported increased abundance of *Blauti*a and *Clostridium Prazmowski* in depressed patients (Cheung et al., 2019). Collectively, these investigations reveal substantial inconsistencies and contradictory findings in current gut microbiota research pertaining to depression.

Given that gut microbiota exhibit distinct compositional characteristics across different life stages, alterations in gut microbial profiles during adolescence may constitute a critical factor mediating the association between stress exposure and depression onset in this developmental period (Freimer et al., 2022). Consequently, several investigations have specifically focused on gut microbiota alterations in adolescent populations. Although Thapa et al. (2021) found no significant group differences in bacterial taxa at phylum and genus levels in their survey of adolescents with depression, Zhou et al. (2023) documented decreased relative abundance of *Faecalibacterium, Roseburia, Collinsella, Blautia, Phascolarctobacterium*, and unclassified *Lachnospiraceae* in adolescent depressed patients, with restoration following sertraline treatment. An investigation comprising 54 healthy controls and 48 adolescents with depression identified 20 differential bacterial taxa participating in eight metabolic pathways, six of which were associated with amino acid metabolism (Tian et al., 2023). Dietary amino acids serve as crucial precursors for neurotransmitter synthesis; specifically, tyrosine, tryptophan, and glutamate function as precursors for dopamine, serotonin (5-HT), and γ-aminobutyric acid (GABA), respectively. Aberrant amino acid metabolism may consequently contribute to depression pathogenesis. To investigate the mechanistic role of gut microbiota in depression regulation, Zhou et al. (2023) performed fecal microbiota transplantation from healthy adolescent donors into adolescent depression-model mice, demonstrating that effective colonization of *Roseburia* significantly elevated 5-HT levels in both brain and colon tissues, thereby ameliorating depressive behaviors. These findings offer promising therapeutic prospects for depression treatment strategies targeting the gut microbiota.

The incidence of adolescent depression continues to escalate, yet therapeutic options for this population remain limited. Compared with adults, various classes of antidepressant medications demonstrate diminished efficacy in adolescents (Kennard et al., 2006). The aforementioned summary of gut microbiota research in depression likely reflects underlying differences in neuropathophysiological mechanisms and gut microbial composition across age groups. Therefore, the present study was designed to collect and comparatively analyze gut microbiota samples from depressed patients across different age groups, with the objective of exploring novel theoretical frameworks and therapeutic directions for depression alleviation and treatment.

## 2 Materials and methods

### 2.1 Subject enrollment criteria

This investigation enrolled a total of 125 participants for gut microbiota analysis, comprising 95 patients diagnosed with depression (33 adolescents aged ≤ 18 years and 62 adults aged 19–80 years) and 30 healthy adolescent controls (aged ≤ 18 years) matched to the adolescent depression cohort. Depression diagnosis was established according to the following comprehensive criteria: (1) fulfillment of diagnostic criteria for depressive disorders as specified in the Diagnostic and Statistical Manual of Mental Disorders, Fifth Edition (DSM-5); (2) Hamilton Depression Rating Scale-17 (HAMD-17) score ≥ 18; (3) unremarkable laboratory examination findings; (4) compliance with pharmacological treatment protocols; (5) and informed consent obtained from patients and/or their legal guardians. Besides, baseline data were collected, including: Demographic characteristics: age, sex, BMI; Lifestyle and behavioral habits: weekly physical exercise frequency, trace element supplementation, multivitamin supplementation, intake frequency of fried food/carbonated drinks/milk tea, dietary taste preference (sweet preference, high-calorie preference); Gastrointestinal symptoms: frequency of constipation and diarrhea; Sleep status: occurrence of difficulty falling asleep, sleep maintenance difficulty (waking up midway), and early awakening; Drug allergy history. All depressed subjects in this study were treatment-naive. None of the participants had taken antidepressants, antibiotics or other medications that affect gut microbiota in the past 3 months. Therefore, confounding factors caused by drug intervention were excluded.

### 2.2 16S rRNA gene sequencing and analysis

Total microbial community DNA was extracted from the fecal samples following the manufacturer's instructions of the E.Z.N.A.^®^ Soil DNA Kit (Omega Bio-tek, Norcross, GA, U.S.). For the analysis of bacterial community structure, PCR amplification was performed using the universal primers for the full-length 16S rRNA gene: 27F (5'-AGRGTTYGATYMTGGCTCAG-3') and 1492R (5'-RGYTACCTTGTTACGACTT-3'). Each amplification primer for the samples contained an eight-base barcode sequence for sample differentiation. Twenty microliters of PCR reaction system was prepared as follows: 4 μL of 5 × FastPfu Buffer, 2 μL of 2.5 mM dNTPs, 0.8 μL each of the forward and reverse primers (5 μM), 0.4 μL of FastPfu Polymerase, and 10 ng of template DNA. The PCR amplification program was set as follows: initial denaturation at 95 °C for 2 min; followed by 25 cycles of denaturation at 95 °C for 30 s, annealing at 55 °C for 30 s, and extension at 72 °C for 1 min; with a final extension at 72 °C for 5 min. The PCR amplicons were separated by 2% agarose gel electrophoresis and then purified using the AxyPrep DNA Gel Extraction Kit (Axygen Biosciences, Union City, CA, U.S.) following the manufacturer's protocol.

SMRTbell libraries were prepared from the amplified DNA via blunt-end ligation in accordance with the manufacturer's instructions (Pacific Biosciences). Each sample was ligated with a specific barcode sequence, and the barcoded amplicons were pooled in equal mass ratios. The amplicon mixture was used to construct sequencing libraries with the Pacific Biosciences SMRTbell™ Template Prep Kit 1.0, and sequencing was conducted on the PacBio Sequel II platform. All amplicon sequencing work was performed by Shanghai Ling'en Biotechnology Co., Ltd. (Shanghai, China). The raw sequence data have been deposited in the Genome Sequence Archive (Genomics, Proteomics & Bioinformatics 2025) in National Genomics Data Center (Nucleic Acids Res 2025), China National Center for Bioinformation/Beijing Institute of Genomics, Chinese Academy of Sciences (GSA: CRA039957) that are publicly accessible at https://ngdc.cncb.ac.cn/gsa.

Raw PacBio reads were processed using SMRT Link Analysis Software (Version 6.0) to generate Circular Consensus Sequence (CCS) reads with the parameters set as follows: minimum pass number = 3 and minimum predicted accuracy = 0.990. Raw reads were further processed via SMRT Portal to filter sequences by length (< 800 bp or > 2,500 bp) and quality. Additional filtering steps were performed to remove barcode and primer sequences, chimeric sequences, and sequences containing 10 consecutive identical bases. Filtered reads were processed using DADA2 (QIIME 2 recommended) to identify insertions, deletions and substitutions, and generate Amplicon Sequence Variants (ASVs). A maximum of two expected errors per read (maxEE = 2) was allowed. After removing chimeric ASV sequences, the phylogenetic affiliations of each 16S rRNA gene sequence (hereafter referred to as ASV) were annotated by the RDP Classifier (https://github.com/rdpstaff/classifier) against the Silva (SSU132) 16S rRNA database with a 70% confidence threshold. Based on the Kyoto Encyclopedia of Genes and Genomes (KEGG) database, PICRUSt2 (http://picrust.github.io/picrust/tutorials/genome_prediction.html) was used to predict functional differences in microbial communities among different samples.

### 2.3 Statistical and bioinformatic analyses

Statistical analyses were conducted using R software version 4.3.0. Continuous variables following normal distribution were expressed as mean ± standard deviation (SD), with between-group comparisons performed using independent samples *t*-test and Wilcoxon rank-sum test. Multiple group comparisons were analyzed using one-way analysis of variance (ANOVA). Categorical variables were presented as *n* (%), with comparisons conducted using Chi-square test or Fisher's exact probability test. Alpha diversity and Beta diversity were assessed by ANCOVA and PERMANOVA with age as a covariate. Differential abundance analysis of microbial taxa was performed using ANCOM-BC2 with age as a covariate, Group × age interaction analysis for microbial abundance was conducted using LinDA. Multiple comparisons were corrected using false discovery rate (FDR) adjustment to eliminate false positive results, with *q* < 0.05 considered statistically significant. Random Forest classifiers was used for model construction and model performance was evaluated using both out-of-bag (OOB) estimates and five-fold cross-validation (5-CV). All experimental figures were generated using GraphPad Prism 8, Origin 2021, and OmicStudio tools (https://www.omicstudio.cn/tool).

## 3 Results

### 3.1 Baseline characteristics

Baseline data serve as critical reference points for evaluating participant status prior to study commencement, providing essential benchmarks for subsequent disease progression monitoring and therapeutic efficacy assessment (Gogishvili et al., 2022). The present investigation enrolled 125 participants, stratified into three distinct cohorts: adolescent depression (Dep-adolescent group), adult depression (Dep-adult group), and healthy adolescent controls (Con-adolescent group). The adolescent depression cohort comprised 33 individuals (26.40%), the adult depression cohort consisted of 62 individuals (49.60%), and 30 healthy adolescents control were matched as controls for the adolescent depression group (24%).

Comparative analysis of baseline characteristics between adolescent and adult depression (Dep-adult) is presented in Table 1. Statistical analysis revealed significant between-group differences in age (*P* < 0.001), while no significant differences were observed in height, weight, body mass index (BMI), or gender distribution (*P* > 0.05). These findings collectively indicate that age should be considered as a covariate when analyzing between-group differences in adolescent vs. adult depression. Beyond these anthropometric and demographic factors, other baseline characteristics such as physical activity (*P* = 1.000), micronutrient supplementation (*P* = 1.000), multivitamin supplementation (*P* = 0.177), and intake of fried food (*P* = 0.200), carbonated drinks (*P* = 0.054), milk tea (*P* = 0.424), constipation (*P* = 0.168) and so on, were showed no statistical differences (all *P* > 0.05). All of them were excluded from subsequent analyses (Supplementary Table S1a).

**Table 1:** Comparison of baseline data between teenager and adult patients with depression.

Variables	Total (*n* = 95)	Dep-adult (*n* = 62)	Dep-adolescent (*n* = 33)	Statistic	*P*
Age	33.53 ± 20.26	44.55 ± 18.64	15.85 ± 1.64	*t* = 11.14	< 0.001
Height (cm)	166.07 ± 8.50	164.57 ± 8.76	167.47 ± 8.31	*t* = −0.91	0.369
Weight (kg)	65.38 ± 18.70	67.21 ± 14.93	63.67 ± 22.04	*t* = 0.50	0.619
BMI	23.73 ± 6.83	24.83 ± 5.44	22.71 ± 7.96	*t* = 0.83	0.414
Gender, *n* (%)				χ^2^ = 2.34	0.126
Male	25 (30.86)	13 (25.00)	12 (41.38)		
Female	56 (69.14)	39 (75.00)	17 (58.62)		

t, t-test; χ^2^, Chi-square test.

Table 2 presents the comparative baseline data between the adolescent depression (Dep-adolescent) and healthy adolescent (Con-adolescent). No statistically significant differences were detected between the depression and healthy groups regarding age, height, weight, BMI, or gender (*P* > 0.05). Additionally, no significant differences were found in dietary preferences, drug allergy history, or other dietary intake (all *P* > 0.05). In contrast, sleep-related indicators (difficulty falling asleep, *P* = 0.012; sleep maintenance difficulty, *P* = 0.052) and gastrointestinal symptoms (constipation, *P* = 0.002; diarrhea, *P* = 0.007), which are well-recognized manifestations of depression, exhibited significant or near-significant differences. Given their nature as depressive symptoms rather than independent confounding factors, these sleep- and gastrointestinal-related indicators were excluded from subsequent analyses. These results confirm that anthropometric, demographic, and dietary variables do not act as significant confounders in the association analysis between depression and gut microbiota, thereby supporting the validity of subsequent theoretical analyses (Supplementary Table S1b).

**Table 2:** Comparison of baseline data between teenager patients with depression and healthy people.

Variables	Total (*n* = 63)	Con-adolescent (*n* = 30)	Dep-adolescent (*n* = 33)	Statistic	*P*
Age	16.02 ± 1.90	16.20 ± 2.17	15.85 ± 1.64	*t* = 0.73	0.469
Height (cm)	167.62 ± 7.85	167.71 ± 7.76	167.47 ± 8.31	*t* = 0.10	0.925
Weight (kg)	58.00 ± 15.83	55.17 ± 10.98	63.67 ± 22.04	*t* = −1.41	0.176
BMI	20.80 ± 5.71	19.81 ± 3.93	22.71 ± 7.96	*t* = −1.33	0.201
Gender, *n* (%)				χ^2^ = 0.01	0.914
Male	24 (40.68)	12 (40.00)	12 (41.38)		
Female	35 (59.32)	18 (60.00)	17 (58.62)		

t, t-test; χ^2^, Chi-square test.

### 3.2 Sequencing metrics

Full-length 16S rRNA gene sequencing generated a total of 4,474,750 high-quality reads, with an average sequencing depth of 35,798 reads per sample and mean read length of 1,492 ± 5 bp. Sequence denoising algorithms (DADA2) were applied for data optimization, yielding 10,527 amplicon sequence variants (ASVs). Taxonomic classification using the RDP classifier Bayesian algorithm assigned these ASVs to 1,757 species-level taxa. However, given that the majority of species-level assignments remained unannotated, genus-level taxonomy was selected for in-depth analytical investigation.

### 3.3 Gut microbial diversity analysis in adolescent depression

**Figure 1:**
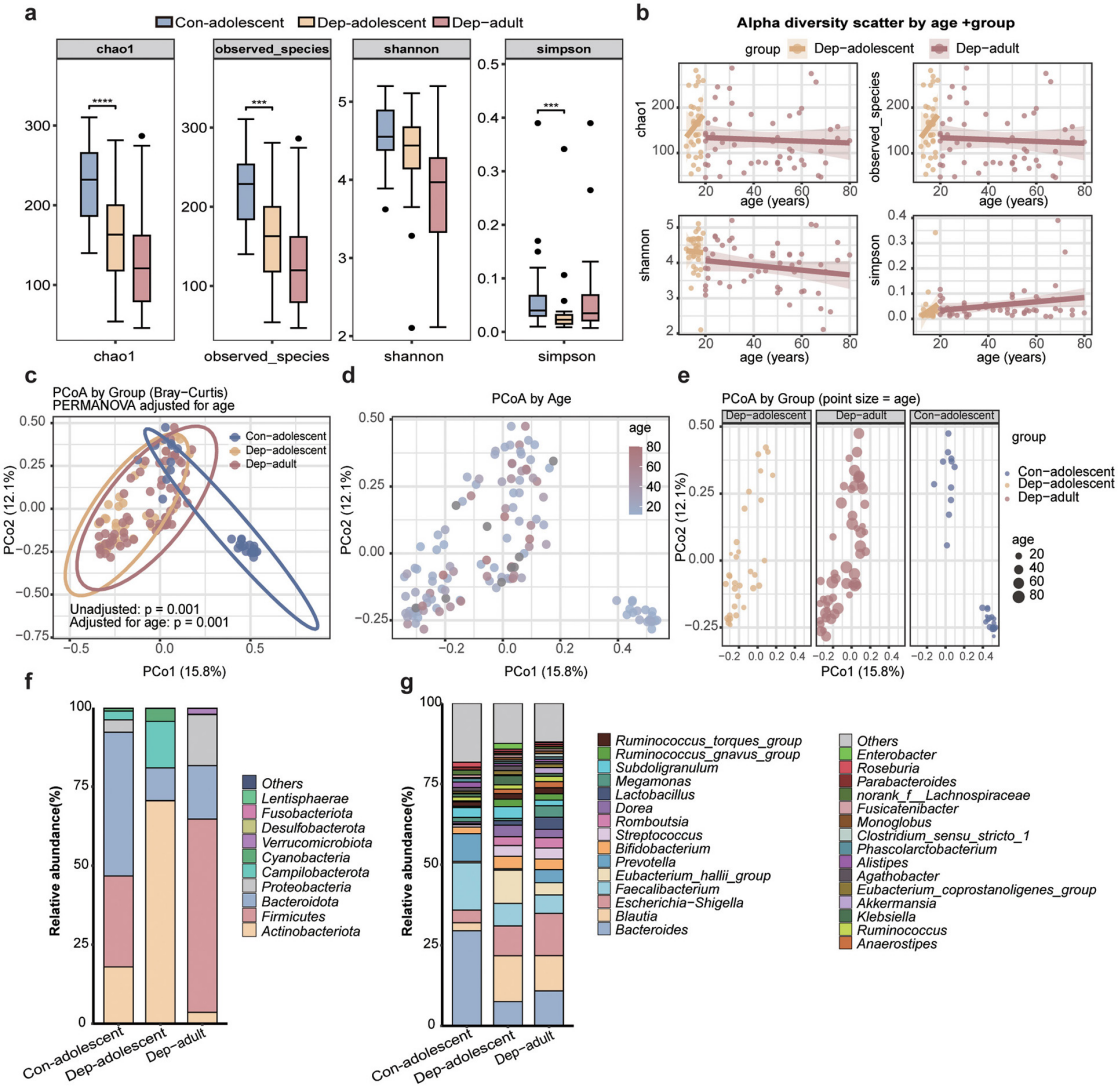
Gut microbial analysis in adolescent depression. **(a)** Box plots showing alpha diversity indices (Chao1, observed species, Shannon, and Simpson) across three groups by ANCOVA with age as a covariate; **(b)** Scatter plots depicting the correlation between age and alpha diversity indices (Chao1, observed species, Shannon, and Simpson) stratified by group (Dep-adolescent and Dep-adult) with regression lines and 95% confidence intervals shown; **(c)** Principal Coordinates Analysis (PCoA) based on Bray-Curtis dissimilarity, PERMANOVA results are shown for both unadjusted and age-adjusted models. **(d)** PCoA plot colored by age to illustrate the effect of age on microbial community structure; **(e)** PCoA plot with point size proportional to age, colored by group, to visualize the interaction between age and group on beta diversity; **(f)** Phylum-level relative abundance comparison across the three cohorts (bar plot); **(g)** Genus-level relative abundance comparison (top 30 genera) across the three cohorts (bar plot). (Blue: Con-teenager, Yellow: Dep-adolescent, Pink: Dep-adult; ***P < 0.001, and ****P < 0.0001).

Alpha diversity indices serve as primary metrics for characterizing gut microbial community diversity, reflecting species richness, community evenness, and overall diversity, encompassing Chao1, Observed_species, Shannon, and Simpson indices. Elevated Chao1 and Observed_species indices indicate greater total species abundance, whereas increased Shannon and Simpson indices reflect enhanced microbial richness and evenness. Our results demonstrated that depressed patients exhibited significantly reduced α-diversity indices compared with healthy adolescent (Con-adolescent). Specifically, the adolescent depression (Dep-adolescent) displayed significantly lower Chao1, Observed_species, and Simpson indices relative to healthy adolescents (Con-adolescent; *P* < 0.001). However, adolescent depression (Dep-adolescent) exhibited no significantly change compared with adult depression (Dep-adult), using ANCOVA with age as a covariate (Figure 1a). Scatter plots examining the correlation between age and alpha diversity within each group revealed no significant associations in either Dep-adolescent or Dep-adult (Figure 1b). These findings indicate that adolescent depression is associated with reduced gut microbiota richness and diversity, a pattern that is not distinct from that observed in adult depression.

Beta diversity analysis quantifies similarities or distances between microbial community pairs (Su, 2021). Beta diversity was evaluated using PCoA based on Bray-Curtis dissimilarity. PERMANOVA adjusted for age confirmed significant separation among the three groups, with results remaining robust after age correction (unadjusted *P* = 0.001; age-adjusted *P* = 0.001; Figure 1c), demonstrating that disease status contributed substantially to microbial community divergence, although age itself also exhibited a significant independent effect (*R*^2^ = 0.010, *F* = 1.730, and *P* = 0.039), and the lack of a healthy adult control precludes complete disentanglement of age vs. disease effects (Supplementary Table S2), and an obvious age gradient was also observed in the PCoA ordination space (Figure 1d).

Further grouping analysis with point size encoding age information (Figure 1e) showed that samples in the adolescent depression group were concentrated at a younger age, the adult depression group had a broad age span, and the healthy control group exhibited a relatively uniform age distribution. Despite distinct age characteristics within each group, the intergroup separation pattern remained robust after age correction. Further testing of the Group × Age interaction showed a significant interaction term (*R*^2^ = 0.040, *F* = 2.770, and *P* = 0.001; Supplementary Table S2), suggesting that the effect of depression on gut microbiota composition may vary by age, though interaction effects alone cannot establish whether this reflects disease-specific modulation of developmental trajectories or normative age-related microbial maturation. These findings are consistent with the presence of alterations in gut microbial composition associated with adolescent depression.

### 3.4 Gut microbial composition analysis in adolescent depression

Phylum-level relative abundance analysis (Figure 1f) revealed that the healthy adolescents (Con-adolescent) was dominated by *Bacteroidota* and *Firmicutes*, with *Actinobacteriota* representing a secondary dominant phylum. The adult depression (Dep-adult) exhibited dominance of *Firmicutes, Proteobacteria*, and *Bacteroidota*. Notably, the adolescent depression (Dep-adolescent) demonstrated significantly reduced relative abundance of *Bacteroidota* and *Firmicutes*, concomitant with significant elevations in *Actinobacteriota, Campilobacterota*, and *Cyanobacteria*. These observations suggest that adolescent depression (Dep-adolescent) is characterized by distinctive phylum-level compositional alterations that differ from both healthy adolescents (Con-adolescent) and adult depression (Dep-adult).

Analysis of the top 30 most abundant genera revealed that, compared with healthy adolescents (Con-adolescent), adolescent depression (Dep-adolescent) exhibited enrichment of *Blautia, Escherichia_Shigella, Eubacterium_hallii_group*, and *Streptococcus* as dominant taxa, while *Bacteroides, Faecalibacterium*, and *Prevotella* demonstrated generally reduced relative abundance. The gut microbial composition of adult depression (Dep-adult) showed partial similarity to that of adolescent depression (Dep-adolescent), yet maintained age-specific differential characteristics (Figure 1g).

### 3.5 Differential gut microorganism analysis in adolescent depression

To identify microbial taxa differentially associated with adolescent depression while adjusting for age-related confounding, differential abundance analysis of 185 genera with mean relative abundance >1‰ (Nikodemova et al., 2024) was performed using ANCOM-BC2 with age as a covariate.

**Figure 2:**
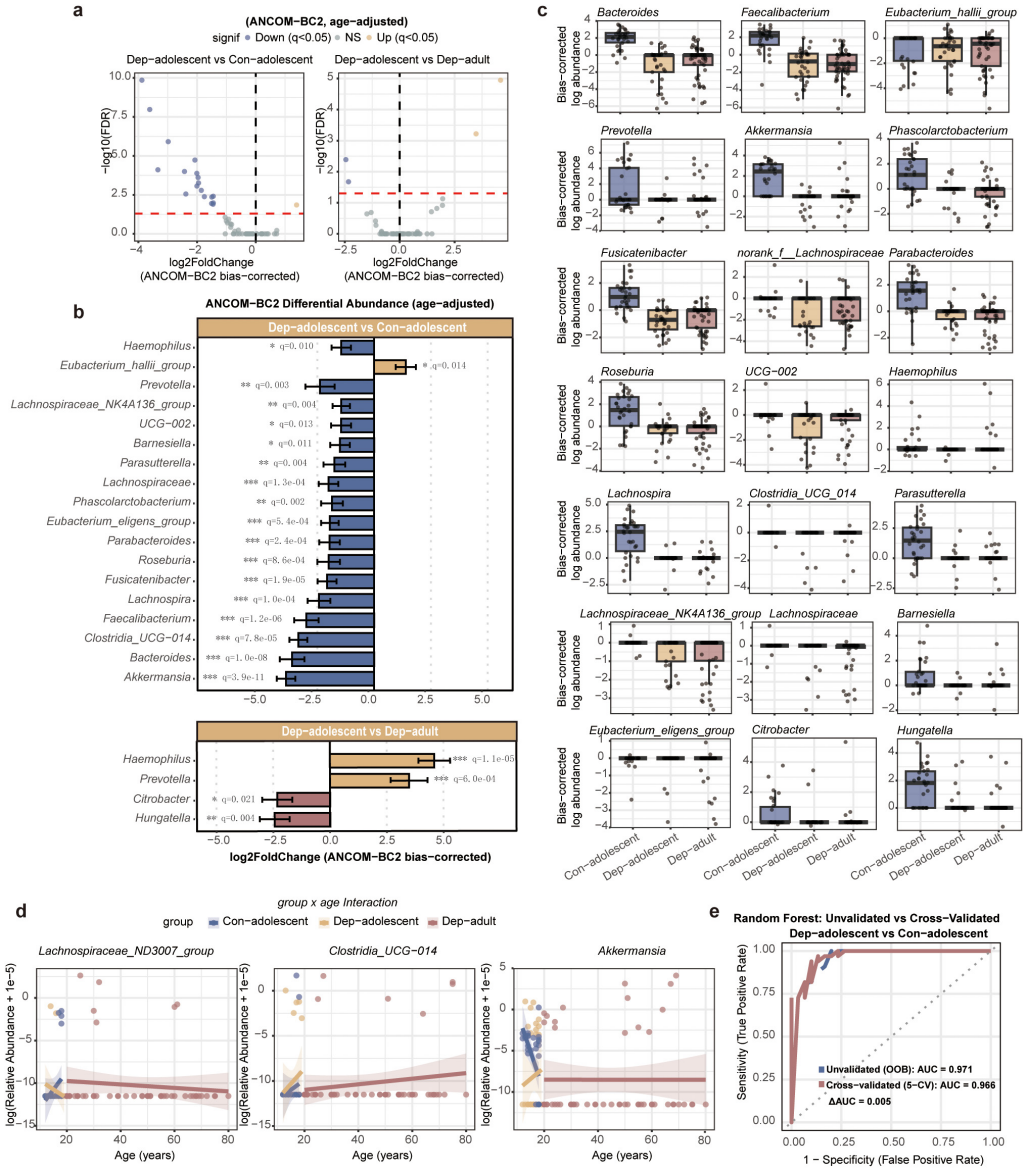
Differential gut microbial composition analysis in adolescent depression. **(a)** Volcano plots showing differentially abundant genera identified by ANCOM-BC2 (age-adjusted); **(b)** Bar plots presenting ANCOM-BC2 differential abundance results (age-adjusted) for significantly altered genera. Bar colors indicate direction of change (blue: downregulated in Dep-adolescent; orange: upregulated in Dep-adolescent). *q < 0.05, **q < 0.01, ***q < 0.001; **(c)** Box plots showing the relative abundance (bias-corrected) of significantly differentially abundant genera across three groups; **(d)** Scatter plots illustrating group × age interaction effects on the relative abundance of three genera (*Lachnospiraceae_ND3007_group, Clostridia_UCG-014*, and *Akkermansia*) identified by interaction analysis; **(e)** Receiver Operating Characteristic (ROC) curves comparing the performance of Random Forest models for discriminating Dep-adolescent from Con-adolescent. The unvalidated model (OOB) achieved an AUC of 0.971, while the five-fold cross-validated model (5-CV) achieved an AUC of 0.966, with a ΔAUC of 0.005, indicating high model stability.

Compared with healthy adolescents (Con-adolescent), the adolescent depression (Dep-adolescent) exhibited significant differential 19 genera after false discovery rate (FDR) correction (*q* < 0.05; Figure 2a). Among these, 18 genera were significantly depleted in Dep-adolescent, including several key genera: *Akkermansia* (*q* = 3.90 × 10^−11^), *Faecalibacterium* (*q* = 1.20 × 10^−6^), *Roseburia* (*q* = 8.60 × 10^−4^), *Lachnospira* (*q* = 1.00 × 10^−4^), *Fusicatenibacter* (*q* = 1.90 × 10^−5^), and *Eubacterium eligens group* (*q* = 5.40 × 10^−4^). Additionally, *Parabacteroides* (*q* = 2.40 × 10^−4^), *Phascolarctobacterium* (*q* = 2.00 × 10^−2^), *Lachnospiraceae NK4A136 group* (*q* = 4.00 × 10^−3^), *Barnesiella* (*q* = 1.10 × 10^−2^), *Parasutterella* (*q* = 4.00 × 10^−4^), were significantly reduced. In contrast, only *Eubacterium hallii group* (*q* = 1.40 × 10^−2^) were significantly enriched in Dep-adolescent (Figure 2b). Comparative analysis between adolescent and adult depression (Dep-adult) identified four significant genera, *Haemophilus* (*q* = 1.10 × 10^−5^) and *Prevotella* (*q* = 6.00 × 10^−4^) were higher in Dep-adolescent, whereas *Citrobacter* (*q* = 0.02) and *Hungatella* (*q* = 4.00 × 10^−3^) were higher in Dep-adult (Figure 2b).

Although Dep-adult group weren't compared to Con-adolescent, the three-group comparison in Figure 2c reveals a conserved core dysbiosis module shared across adolescent and adult depression. Among the 21 key genera identified by ANCOM-BC2, 16 genera (including *Bacteroides, Faecalibacterium, Prevotella, Akkermansia, Roseburia*, and *Parasutterella*) showed concordant depletion in both Dep-adolescent and Dep-adult groups relative to Con-adolescent controls. Notably, these genera exhibited no significant differences between the two depressed groups (Dep-adolescent vs. Dep-adult, *q* > 0.05), suggesting that their depletion may represent a shared feature of depression-associated gut microbiota alteration across age groups (Figure 2c). Notably, *Prevotella* and *Haemophilus* exhibited a distinct age-dependent depletion pattern: it was significantly reduced in Dep-adolescent compared to Con-adolescent (*q* < 0.05), and further significantly depleted in Dep-adult (*q* < 0.05), forming a progressive depletion gradient (Dep-adult < Dep-adolescent < Con-adolescent). Conversely, *Citrobacter* and *Hungatella* displayed strictly adult-specific enrichment characteristics: no significant differences were observed between adolescent depression and healthy controls, but both were significantly elevated in Dep-adult (*q* < 0.05). This suggests that the enrichment of these two genera may represent a feature more prominent in adult depression.

To investigate whether the relationship between age and microbial abundance differs by disease status, we performed group × age interaction analysis. Three genera exhibited significant interaction effects (Figure 2d): *Lachnospiraceae ND3007 group, Clostridia UCG-014*, and *Akkermansia*. In healthy adolescents, the abundances of these taxa remained relatively stable or declined modestly with age, whereas in depressed adolescents, they showed marked age-related decreases, ultimately reaching levels comparable to those in depressed adults. These findings suggest that depression may disrupt the normal developmental trajectory of the gut microbiota, leading to premature acquisition of an “adult-like” dysbiotic profile in affected adolescents.

### 3.6 Diagnostic model analysis of key biomarker genera

To further construct auxiliary diagnostic models for depression and age-stratified depression, the 19 differential bacterial taxa specific to adolescents, as well as four differential bacteria and three interactive bacteria identified in the depressed group, were screened for subsequent ROC curve analysis. Single-genus ROC analysis for discriminating Dep-adolescent from Con-adolescent (Supplementary Figure S1a) revealed limited diagnostic utility of individual taxa, with AUC values ranging from 0.423 (*Eubacterium eligens group*) to 0.885 (*Eubacterium hallii group*). To construct robust diagnostic models, Random Forest classifiers were developed using the differentially abundant genera AUC > 0.700. For Dep-adolescent vs. Con-adolescent (Figure 2e), the unvalidated model (out-of-bag, OOB) achieved an AUC of 0.971, while five-fold cross-validation (5-CV) yielded an AUC of 0.966 (Sensitivity = 1.000, Specificity = 0.800, Accuracy = 0.905, Precision = 0.846, *F*1 = 0.917), with a minimal Δ AUC of 0.005. The near-identical performance between OOB and cross-validated estimates suggest potential stability in internal validation but requires external validation.

For Dep-adolescent vs. Dep-adult, Single-genus ROC analysis for Dep-adolescent vs. Dep-adult (Supplementary Figure S1b) revealed poor discriminatory capacity (AUC range: 0.471–0.551). The Random Forest model performed substantially worse: OOB AUC = 0.625, 5-CV AUC = 0.548 (Sensitivity = 0.333, Specificity = 0.774, Accuracy = 0.379, Precision = 0.314, *F*1 = 0.324), with a Δ AUC of 0.077 (Supplementary Figure S1c). The marked drop in cross-validated performance and the AUC approaching 0.500 (random chance) indicate that gut microbiota features cannot reliably distinguish adolescent from adult depression.

### 3.7 Metabolic function predictions in adolescent depression

**Figure 3:**
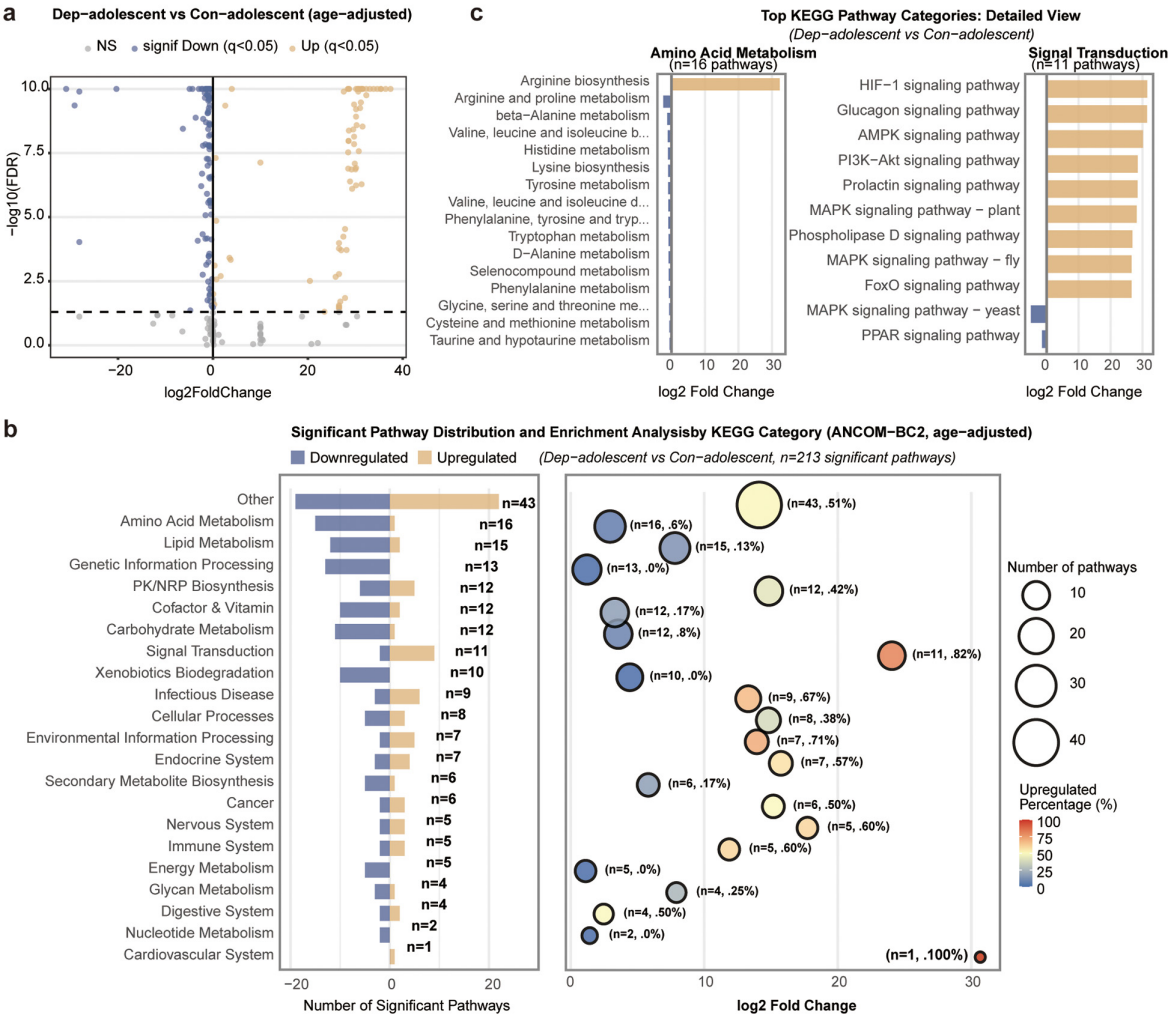
Functional prediction and metabolic pathway analysis of gut microbiota in adolescent depression. **(a)** Volcano plot showing differentially abundant KEGG pathways between Dep-adolescent and Con-adolescent identified by Linear Model with age as a covariate, each point represents a metabolic pathway, with color indicating significance status: blue (downregulated, *q* < 0.05), yellow (upregulated, *q* < 0.05), and gray (non-significant, NS); **(b)** Significant pathway distribution and enrichment analysis by KEGG category. Left panel: stacked bar chart showing the number of significantly upregulated (yellow) and downregulated (blue) pathways across functional categories. Right panel: bubble plot displaying the enrichment characteristics of each category, with bubble size representing the number of pathways, color intensity reflecting the percentage of upregulated pathways within each category. **(c)** Detailed view of the top two KEGG pathway categories, Left: Amino Acid Metabolism (*n* = 16 pathways); Right: Signal Transduction (*n* = 11 pathways; blue: downregulated; yellow: upregulated).

PICRUSt2 was employed for metabolic functional prediction based on 16S rRNA gene sequences. Differential analysis was performed using Linear Model with age as a covariate. A total of 213 significantly differential metabolic pathways were identified between adolescent depression group and adolescent control group (FDR-corrected *q* < 0.05), including 77 upregulated pathways and 137 downregulated pathways (Figure 3a). No differential metabolic pathways were found between adolescent depression group and adult depression group, suggesting that metabolic differences between these two depressed groups may be less pronounced than those between depressed and healthy adolescents.

KEGG predicted functional enrichment analysis revealed 18 enriched categories (Figure 3b). Amino Acid Metabolism was the most differential pathways (*n* = 16) and Signal Transduction was the highest mean effect size pathway (mean |log_2_FC| = 24.80; Figure 3c). In Amino Acid Metabolism, Dep-adolescent group showed synthetic-degradative imbalance: Arginine biosynthesis was markedly upregulated (log_2_FC = +32.30, *q* = 2.69 × 10^−12^) whereas Arginine and proline metabolism was downregulated (log_2_FC = −2.43, *q* = 3.69 × 10^−12^). BCAA metabolism was bidirectionally disrupted with upregulated biosynthesis but downregulated degradation, suggesting BCAA accumulation. Tryptophan metabolism was downregulated (log_2_FC = −0.86, *q* = 2.08 × 10^−4^), consistent with the tryptophan-kynurenine axis hypothesis in depression. In Signal Transduction, energy-stress pathways were activated (HIF-1 signaling pathway: log_2_FC = +31.48, *q* = 1.78 × 10^−11^; Glucagon signaling pathway: log_2_FC = +31.43, *q* = 4.83 × 10^−12^; AMPK signaling pathway: log_2_FC = +30.16, *q* = 2.24 × 10^−10^), whereas PPAR signaling pathway, a key regulator of fatty acid oxidation and anti-inflammatory response, was significantly downregulated (log_2_FC = −1.45, *q* = 5.92 × 10^−16^). Together, these predicted findings indicate that gut microbiota metabolic dysfunction in adolescent depression involves coordinated disturbances in amino acid neurotransmitter precursors, energy production, and inflammatory signaling, potentially contributing to depression pathophysiology through the gut-brain axis.

Gut-brain axis pathways showed marked neurotransmitter metabolic dysregulation: Glutamatergic synapse (log_2_FC = +29.90, *q* = 4.04 × 10^−8^) and GABAergic synapse (log_2_FC = +30.03, *q* = 6.97 × 10^−8^) were significantly upregulated, whereas D-Glutamine and D-glutamate metabolism (log_2_FC = −1.11, *q* = 7.33 × 10^−14^).

Energy metabolism was broadly impaired, with Oxidative phosphorylation (log_2_FC = −1.06, *q* = 1.40 × 10^−13^), Citrate cycle (log_2_FC = −1.10, *q* = 2.39 × 10^−13^) and Glycolysis/Gluconeogenesis (log_2_FC = −0.59, *q* = 7.75 × 10^−13^) all significantly downregulated. Short-chain fatty acid metabolism was also compromised, with Butanoate metabolism (log_2_FC = −0.49, *q* = 1.80 × 10^−8^) and Propanoate metabolism (log_2_FC = −0.33, *q* = 7.46 × 10^−5^) both significantly downregulated, suggesting impaired SCFA production capacity. Bile acid metabolism showed synthetic-conversion imbalance: Primary bile acid biosynthesis was significantly downregulated (log_2_FC = −1.48, *q* = 1.98 × 10^−9^), whereas Secondary bile acid biosynthesis was upregulated (log_2_FC = +0.36, *q* = 7.74 × 10^−4^), indicating altered bile acid pool composition and microbial biotransformation activity.

Endocrine and immune pathways were also altered, including downregulated Adipocytokine signaling pathway (log_2_FC = −2.17, *q* = 2.32 × 10^−14^) and Insulin signaling pathway (log_2_FC = −0.81, *q* = 7.97 × 10^−11^)), and upregulated Insulin resistance (log_2_FC = +30.16, *q* = 8.23 × 10^−12^), IL-17 signaling pathway (log_2_FC = +28.58, *q* = 4.66 × 10^−9^) and Th17 cell differentiation (log_2_FC = +28.49, *q* = 1.41 × 10^−8^).

#### 3.7.1 Differential enzyme analysis of neurotransmitter-related amino acid metabolism

Given the established association between tryptophan metabolism dysfunction and depression pathogenesis, and the central role of amino acid metabolism in neurotransmitter biosynthesis and gut-brain axis communication, we further investigated predicted enzyme-level alterations in three key neurotransmitter-related pathways: tryptophan, tyrosine, and glutamate-GABA metabolism.

**Figure 4:**
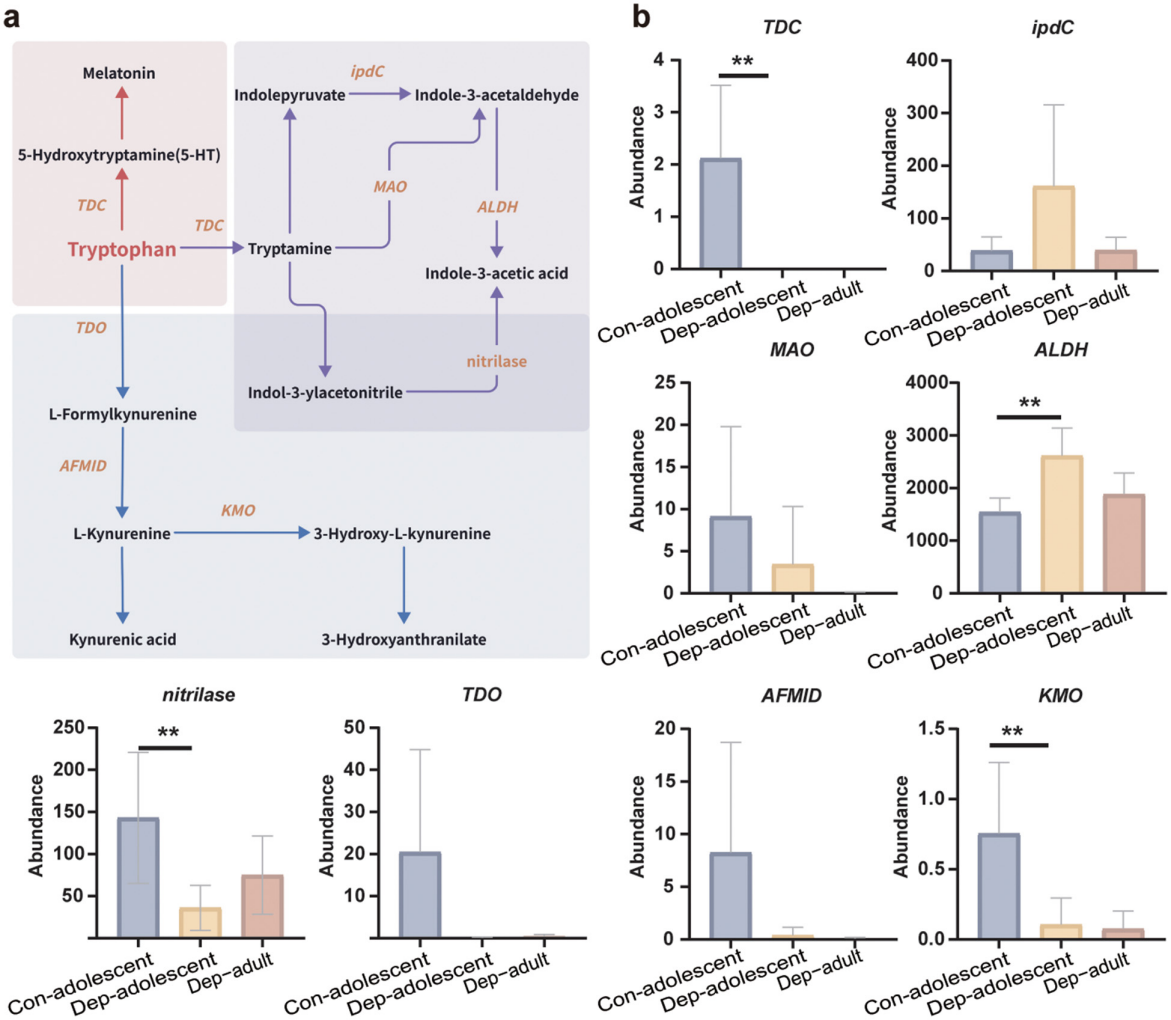
Differential analysis of tryptophan metabolism enzymes. **(a)** Tryptophan metabolism pathways; **(b)** Differential analysis of key enzymes in tryptophan metabolism pathways across the three cohorts [*TDC*, tryptophan decarboxylase; *TDO*, kynurenine pathway enzymes tryptophan 2,3-dioxygenase; *AFMID*, arylformamidase; *KMO*, kynurenine 3-monooxygenase; *ipdC*, indolepyruvate decarboxylase; *MAO*, monoamine oxidase; *ALDH*, aldehyde dehydrogenase (NAD+). Blue: Con-teenager, Yellow: Dep-adolescent, and Pink: Dep-adult; **P < 0.01].

Tryptophan−5-HT: Compared with healthy adolescents, adolescent depression showed significantly altered predicted activities of tryptophan decarboxylase (*TDC*) and aldehyde dehydrogenase (NAD^+^) (*ALDH*). Notably, no significant differences were detected between adolescent and adult depression for any of the above tryptophan metabolic enzymes, suggesting that alterations in tryptophan metabolic enzymes are not age-specific (Supplementary Figure S2a; Figure 4).

**Figure 5:**
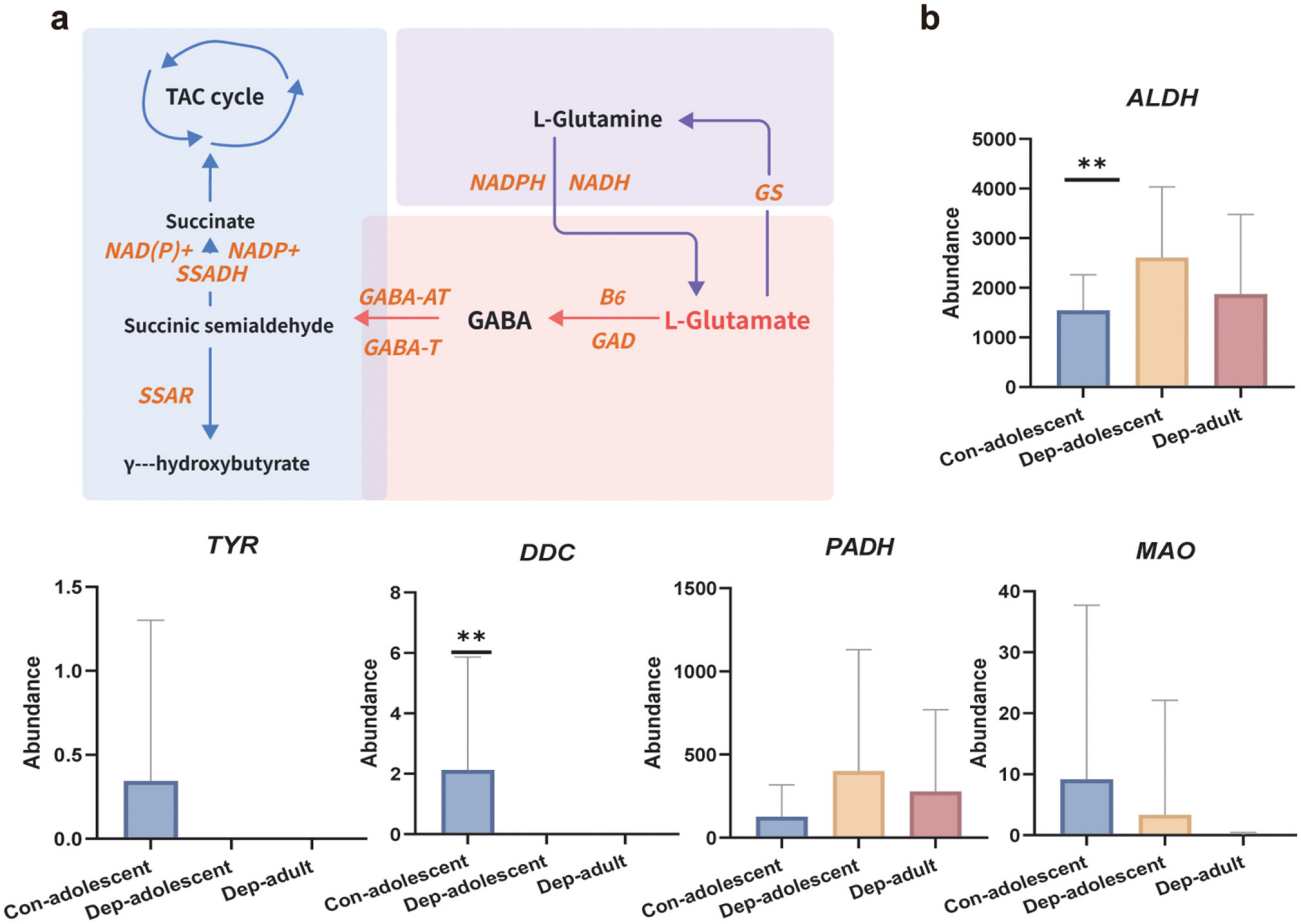
Differential analysis of tyrosine metabolism enzymes. **(a)** Tyrosine metabolism pathways; **(b)** Differential analysis of key enzymes in tyrosine metabolism pathways across the three cohorts (*ALDH*, aldehyde dehydrogenase; *TYR*, tyrosinase; *DDC*, dopa decarboxylase; *PADH*, phenylacetaldehyde dehydrogenase; *MAO*, monoamine oxidase. Blue: Con-teenager, Yellow: Dep-adolescent, Pink: Dep-adult; **P < 0.01).

Tyrosine–dopamine: Tyrosine metabolism mediates dopamine and norepinephrine synthesis (Supplementary Figure S2b; Figure 5). Compared with healthy adolescents, adolescent depression exhibited significantly altered predicted activities of aldehyde dehydrogenase (*ALDH*) and dopa decarboxylase (*DDC*). No significant differences in tyrosine metabolism-related enzymes were found between adolescent and adult depression.

**Figure 6:**
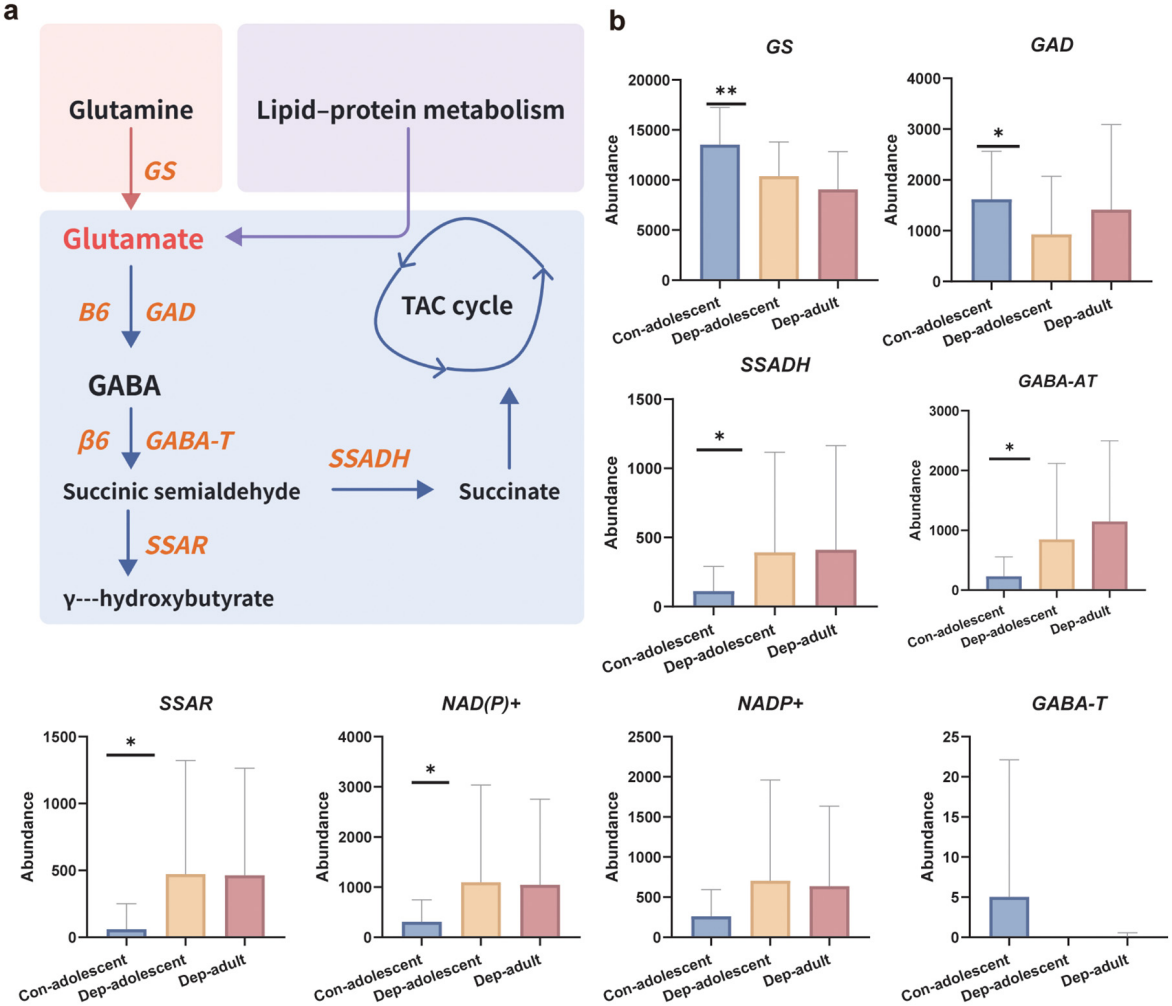
Differential analysis of alanine, aspartate, and glutamate metabolism enzymes. **(a)** Alanine, aspartate, and glutamate metabolism pathways; **(b)** Differential analysis of key enzymes in Alanine, aspartate, and glutamate metabolism pathways across the three cohorts. (*GS*, glutamine synthetase; *GABA-AT*, 4-aminobutyrate-2-oxoglutarate transaminase; *GABA-T*, 4-aminobutyrate-pyruvate transaminase; *GAD*, glutamate decarboxylase; *SSADH*, succinic semialdehyde dehydrogenase; *SSAR*, succinic semialdehyde reductase. Blue: Con-teenager, Yellow: Dep-adolescent, Pink: Dep-adult; *P < 0.05, **P < 0.01).

Glutamate–GABA metabolism: The glutamate-GABA shunt pathway balances excitatory-inhibitory neurotransmission (Supplementary Figure S2c; Figure 6). Compared with healthy adolescents, adolescent depression showed significantly altered predicted abundances of glutamine synthetase (*GS*), 4-aminobutyrate-2-oxoglutarate transaminase (*GABA-AT*), succinic semialdehyde reductase (*SSAR*), glutamate decarboxylase (*GAD*), and NAD(P)^+^-related enzymes. No significant differences in glutamate-GABA metabolism-related enzymes were detected between adolescent and adult depression.

Collectively, these predicted enzymatic analyses indicate that adolescent depression exhibits specific alterations at the enzyme level in neurotransmitter-related amino acid metabolism. However, these changes showed no significant differences between adolescent and adult depression, suggesting that these metabolic enzyme alterations may represent common features of depression rather than age-specific biomarkers.

## 4 Discussion

Adolescent depression represents a distinct clinical subtype of depressive disorders, characterized by ongoing neuro development, heightened emotional reactivity, and complex interactions between environmental exposures and biological vulnerability. These features distinguish adolescent depression from adult depression not only at the psychological level but also in biological regulation, including gut–brain axis interactions (Wu et al., 2026). It is inappropriate to simply extrapolate the pathological characteristics of adult depression to adolescents. Taking advantage of this critical window of physiological development, this study adopted full-length 16S rRNA gene sequencing to characterize gut microbiota dysbiosis in drug-naive adolescents with depression. The results confirmed that adolescent depression presents distinctive microbial dysbiosis, including reduced alpha diversity, significant structural remodeling in the abundance of 19 genera, and obvious perturbations in amino acid metabolism and signal transduction pathways. Meanwhile, four genera showed significant differences between adolescent and adult depression. By establishing three comparative groups—adolescent depression, healthy adolescents, and adult depression—this study attempted to differentiate microbial signatures associated with adolescent depression from those related to age, filling an important methodological gap in current research (Gao et al., 2023; Winter et al., 2018). Further inclusion of healthy adults as a control group would strengthen the significance of future studies.

The theoretical framework of this study is based on the developmental matching principle: the adolescent gut microbiome remains in an immature stage, which renders it more vulnerable to depression-induced microbial dysbiosis and also provides a valuable window for clinical intervention (Campisi et al., 2025; Flannery et al., 2019). This view is consistent with contemporary neuropsychiatric consensus: the pathophysiological mechanisms of early-onset depression are distinctly different from those of adult depression, predominantly involving neurodevelopmental processes and neuroimmune sensitization during critical developmental windows (Li et al., 2026). Without timely intervention, adolescent depression may lead to irreversible alterations in neurodevelopmental trajectories (Kessler et al., 2010; Thapar et al., 2012).

Our results revealed that adolescents with depression exhibited significantly reduced alpha diversity compared with healthy controls, which supports the gut dysbiosis hypothesis of depression. Reduced microbial diversity impairs functional redundancy, weakens colonization resistance against opportunistic pathogens, and diminishes the capacity to synthesize neuroactive metabolites essential for central nervous system homeostasis (Valles-Colomer et al., 2019; Foster and McVey Neufeld, 2013). A consistent magnitude of decline in alpha diversity was observed when comparing depressed adolescents and depressed adults with healthy counterparts. Two plausible explanations are proposed: first, adolescent depression may represent an intermediate stage of microbial impairment that has not yet progressed to the complete diversity depletion seen in chronic adult depression, consistent with the shorter disease course and younger age of adolescent patients (Zhou et al., 2023). Second, the adolescent gut microbiome retains developmental plasticity, enabling partial compensation for depression-associated microbial loss through continuous colonization and environmental microbial supplementation, thereby maintaining certain ecological resilience (Bokulich et al., 2016; Johnson et al., 2019).

The significant reduction in *Bacteroidota* and *Firmicutes* relative abundance observed in adolescent depressed patients aligns with the conclusions of Cao et al.'s (2025) systematic review of gut microbiota in depression. As core phyla within the gut microbiota, *Bacteroidota* participate in host nutrient metabolism and immune system development, while *Firmicutes* represent the primary phylum for short-chain fatty acid (SCFA) synthesis, regulating central nervous system inflammatory responses and neurotransmitter synthesis through the microbiota-gut-brain axis (Zhang et al., 2025; Wu et al., 2026). The concurrent reduction of both phyla directly compromises gut microecological homeostasis. The gut microbiota undergoes age-dependent maturation, transitioning from an early-life profile dominated by *Actinobacteriota* and *Proteobacteria* to an adult configuration where *Firmicutes* and *Bacteroidota* predominate. By age three, the microbiota largely resembles that of adults, but adolescence remains a critical period for fine-tuning, with taxa such as *Bifidobacterium* still differing in abundance from adult levels (Cheng et al., 2025). Here, we found that depressed adolescents (Dep-adolescent) exhibited significantly reduced *Firmicutes* and abnormally elevated *Actinobacteriota*, reflecting a specific disruption of microbiota maturation by adolescent depression. Adolescence is a pivotal stage for HPA axis development and stress sensitization. Chronic stress is associated with sustained cortisol elevation, which may inhibit the growth of certain beneficial *Firmicutes* (e.g., *Faecalibacterium, Roseburia*, and *Ruminococcus*) sensitive to glucocorticoids (Toader et al., 2024). As key producers of anti-inflammatory, barrier-protective short-chain fatty acids (SCFAs), depletion of these butyrate-producing *Firmicutes* may be related to the pro-inflammatory milieu of adolescent depression. Concurrently, shifts in the intestinal microenvironment (e.g., pH) may contribute to the expansion of inflammation-tolerant *Actinobacteriota* (e.g., *Streptococcus*) and *Proteobacteria* (Cheng et al., 2025).

In healthy adults, the microbiota is typically stable, with *Firmicutes* dominant, followed by *Bacteroidota*, and low abundances of *Actinobacteriota* and *Proteobacteria*. Unlike depressed adolescents, depressed adults in this study showed *Firmicutes* enrichment and *Actinobacteriota* depletion, indicating distinct pathophysiological mechanisms between the two populations. Chronic HPA hyperactivity increases cortisol, altering intestinal permeability and mucus secretion to favor stress-tolerant, metabolically efficient *Firmicutes* (Toader et al., 2024). Notably, *Proteobacteria* increased in both depressed groups. As Gram-negative bacteria, their LPS acts as a potent immune activator, potentially sustaining low-grade endotoxemia and chronic inflammation (Cheng et al., 2025). While *Bacteroidota* maintained moderate abundance in both cohorts, within-phylum shifts from commensal to pro-inflammatory lineages may occur (Eribo et al., 2022), despite its inherent functional redundancy under healthy conditions (Moya and Ferrer, 2016).

At the genus level, the most prominent finding was significant depletion of *Faecalibacterium, Akkermansia, Roseburia*, and multiple members of the *Lachnospiraceae* family, including *Lachnospira* and *Fusicatenibacter*. As the only well-characterized species within *Faecalibacterium, Faecalibacterium prausnitzii* is the most abundant butyrate-producing bacterium in the human colon. It exerts potent anti-inflammatory effects through multiple pathways (Sokol et al., 2008; Miquel et al., 2015) and upregulates tight junction protein expression via GPR43 signaling, protecting intestinal barrier integrity and reducing neuronal injury induced by circulating lipopolysaccharide (Peng et al., 2009; Braniste et al., 2014; Erny et al., 2015). These findings suggest that *Faecalibacterium prausnitzii* may serve as a cross-disease gut health biomarker closely linked to neurological function. As a core intestinal mucus-degrading bacterium, *Akkermansia muciniphila* deficiency increases intestinal permeability, exacerbates neuroinflammation, and induces depressive-like behaviors; supplementation with live bacteria or its outer membrane protein *Amuc_1100* can reverse these abnormalities (Everard et al., 2013; Plovier et al., 2017). Notably, *Prevotella* emerged as a ignificantly depleted in adolescent depression, and exhibited a distinct age-dependent depletion gradient (Dep-adult < Dep-adolescent < Con-adolescent), indicating that its reduction may reflect both disease-associated changes and age-related microbial developmental patterns. *Prevotella* is a core member of the human gut microbiota, particularly abundant in individuals consuming fiber-rich diets, and plays critical roles in polysaccharide degradation, short-chain fatty acid (SCFA) production, and maintenance of mucosal immune homeostasis. Recent large-scale microbiome studies have consistently identified *prevotella* depletion as a hallmark of microbial dysbiosis across multiple disease including major depressive disorder (MDD) and Parkinson's disease (Xiong et al., 2018; Cao et al., 2025). In depression specifically, systematic reviews and meta-analyses have documented reduced *prevotella* in multiple independent studies, establishing it as one of the most reproducible microbial alterations in this disorder (Cao et al., 2025). Furthermore, a recent large-sample investigation (Zhu et al., 2026) has provided robust evidence for the diagnostic and mechanistic value of *prevotella* in disease-associated microbiome alterations, underscoring its potential as a translational biomarker. In the context of adolescent depression, The premature loss of *prevotella* in depressed adolescents thus reflect a dual pattern: a potential depression-associated microbial alteration and an age-related developmental change. Whether this represents disrupted developmental programming or alternative maturation trajectories cannot be determined without a healthy adult control.

Several consistent microbial alterations were shared between adolescent and adult depression, forming a core age-invariant dysbiosis module. Depletion of *Bacteroides, Faecalibacterium, Prevotella, Akkermansia, Roseburia*, and *Parasutterella* showed identical alteration patterns in our adolescent cohort and previous adult depression studies. These non-age-specific signatures reflect shared pathogenic mechanisms across the lifespan, including chronic stress-induced HPA axis dysregulation, systemic low-grade inflammation, and intestinal barrier impairment (Sudo et al., 2004; Huang et al., 2025). The conservation of microbial profiles across developmental stages suggests that such changes may be associated with depression pathogenesis (Cryan and Dinan, 2012).

Nevertheless, age-specific microbial signatures distinguishing adolescent from adult depression are equally important. Significant group-by-age interaction effects were observed for *Lachnospiraceae ND3007 group, Clostridia UCG-014*, and *Akkermansia*. Microbial developmental trajectories diverged between healthy and depressed adolescents, with the microbiota of depressed adolescents displaying premature aging characteristics that gradually converge toward the microbial phenotype of adult depression (Thapa et al., 2021; Zhou et al., 2023). This suggests that adolescent depression disrupts the normal maturation process of the gut microbiome, prematurely driving an adult-like dysbiotic profile. This concept of microbial premature aging aligns with findings on early-life adversity, which demonstrate that childhood stress can induce epigenetic age acceleration across multiple tissues (Zannas et al., 2015).

The Random Forest diagnostic model constructed based on differential genera achieved promising exploratory performance in distinguishing depressed adolescents from healthy controls (AUC = 0.966), but poor discriminatory power between adolescent and adult depression (AUC = 0.548). These results are preliminary consistent with a hierarchical model of gut microbial alterations in depression while indicating that microbial composition alone is insufficient for age-stratified diagnosis. The translational application of this diagnostic model must acknowledge methodological limitations. Although the model exhibited low overfitting risk with limited sample sizes (33 depressed adolescents and 30 healthy controls), insufficient model stability requires caution. Nonetheless, the high AUC values and biological plausibility of the biomarkers provide a solid foundation for prospective validation in larger longitudinal cohorts (Collins et al., 2021).

PICRUSt2 prediction revealed that adolescents with depression exhibited significant downregulation of neurotransmitter-related amino acid metabolism, as well as energy metabolism and mitochondrial function pathways including oxidative phosphorylation, tricarboxylic acid cycle, glycolysis/gluconeogenesis, and short-chain fatty acid metabolism. Among these pathways, amino acid metabolism acts as a core hub of the microbiota-gut-brain axis (Xue et al., 2023; Gao et al., 2020; Yang et al., 2020). Specifically, tryptophan metabolism, tyrosine metabolism, and glutamate metabolism were all suppressed, which correspond to the biosynthetic pathways of 5-HT, dopamine, and GABA, respectively. The predicted reductions in energy metabolism and mitochondrial function indicate widespread impairment of microbial metabolic capacity, which may further disturb host mitochondrial activity. According to the mitochondrial hypothesis of depression, cellular energy metabolic dysfunction can trigger neuronal dysfunction, neuroinflammation, and impaired neural plasticity.

In summary, this investigation suggests that adolescent major depression presents a characteristic gut microbiota dysbiosis phenotype, including reduced microbial alpha diversity, depletion of key butyrate-producing genus such as *Faecalibacterium* and *Akkermansia*, and downregulated predicted metabolic functions of gut microbiota, although further external validation is still required. Additionally, the *Lachnospiraceae ND3007 group, Clostridia UCG-014 group*, and *Akkermansia* exhibited age-specific dysbiotic patterns. Gut microbial dysbiosis in depression encompasses both a conserved core module across ages and adolescent-specific microbial signatures, supporting a hierarchical dysbiosis model and providing theoretical evidence for biomarker development and age-stratified precise treatment. The combined microbial biomarkers show excellent diagnostic efficiency in distinguishing depressed adolescents from healthy controls. Combined with the high plasticity of the gut microbiota during adolescence, microbiota-targeted strategies are expected to become an emerging research direction for precision psychiatry in adolescent mental health (Cryan et al., 2019).

These results provide providing preliminary insights for understanding microecological alterations in adolescent depression, while establishing a research foundation for further elucidating the regulatory mechanisms of the microbiota-gut-brain axis in adolescent psychiatric disorders. Despite the strengths of the present study, several limitations should be acknowledged. First, this study was conducted in a single center with a relatively modest sample size, which may limit the generalizability of the findings. Although strict inclusion criteria were applied and demographic characteristics were comparable between groups, larger multi-center cohorts are required to validate the robustness of the observed microbial signatures. Besides, lacking healthy adult controls fails to establish age-related microbiota baselines, making it hard to tell if microbial shifts in adult depression are disease-specific or age-related normal changes. Second, the cross-sectional design precludes causal inference between gut microbiota alterations and adolescent depression. It remains unclear whether the observed microbial dysbiosis contributes to the development of depressive symptoms or represents a consequence of the disorder. Longitudinal studies are needed to clarify the temp oral relationship between microbiota changes and depressive symptom progression. Third, the present study was solely based on fecal microbiome analysis without blood sample collection, which provides taxonomic resolution but limited functional insight, endocrine/hormonal profiles could not be directly assessed. Future studies integrating metagenomic sequencing, metabolomics, and host immune profiling would provide a more comprehensive understanding of microbiota–host interactions in adolescent depression. Finally, although several microbial genera demonstrated strong discriminative ability in the diagnostic model. But feature selection in this study was based on full-sample differential analysis, although five-fold CV was used during model training, potential information leakage risk remains. Due to sample size limitations, nested CV inner training subsamples were insufficient causing severe overfitting and extremely poor generalization performance. The current findings are exploratory and require independent external validation in larger cohorts to confirm clinical translational value.

## Data Availability

The datasets presented in this study can be found in online repositories. The names of the repository/repositories and accession number(s) can be found in the article/Supplementary material.
