# Supplementary material for: Gut microbiota dysbiosis in adolescent depression: a comparative analysis with adult depression and healthy adolescent
Source: Front Microbiol. 2026 Jul 9;17:1849097. doi: 10.3389/fmicb.2026.1849097 (PMC13393218; doi:10.3389/fmicb.2026.1849097)
Supplement: Supplementary file 1 [file Data_Sheet_1.pdf]

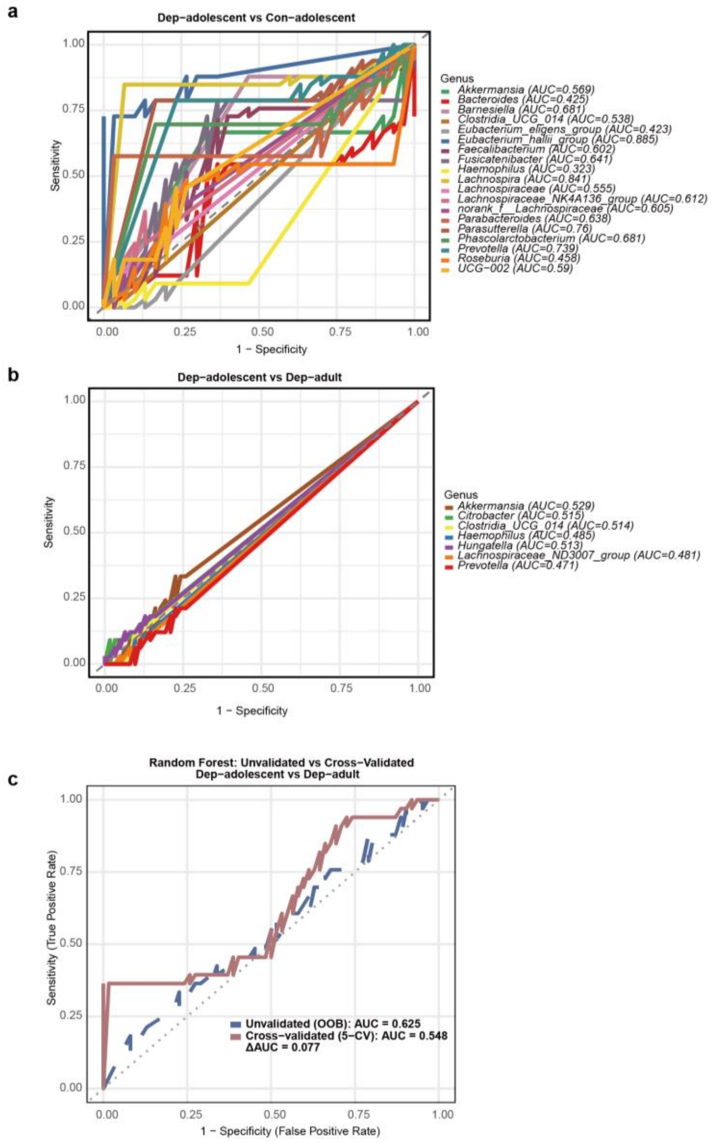

Figure S1 Single-genus ROC curves and Random Forest model comparison for Dep-adolescent vs Dep-adult.

(a) Receiver Operating Characteristic (ROC) curves of individual genera for discriminating Dep-adolescent from Con-adolescent; **(b)** Receiver Operating Characteristic (ROC) curves of individual genera for discriminating Dep-adolescent from Dep-adult; **(c)** Comparison of Random Forest model performance for discriminating Dep-adolescent from Dep-adult. The unvalidated model (OOB) achieved an AUC of 0.625, while the 5-fold cross-validated model (5-CV) achieved an AUC of 0.548, with a Δ AUC of 0.077.
