# Supplementary material for: Gut microbiota dysbiosis in adolescent depression: a comparative analysis with adult depression and healthy adolescent
Source: Front Microbiol. 2026 Jul 9;17:1849097. doi: 10.3389/fmicb.2026.1849097 (PMC13393218; doi:10.3389/fmicb.2026.1849097)
Supplement: Supplementary file 2 [file Data_Sheet_2.pdf]

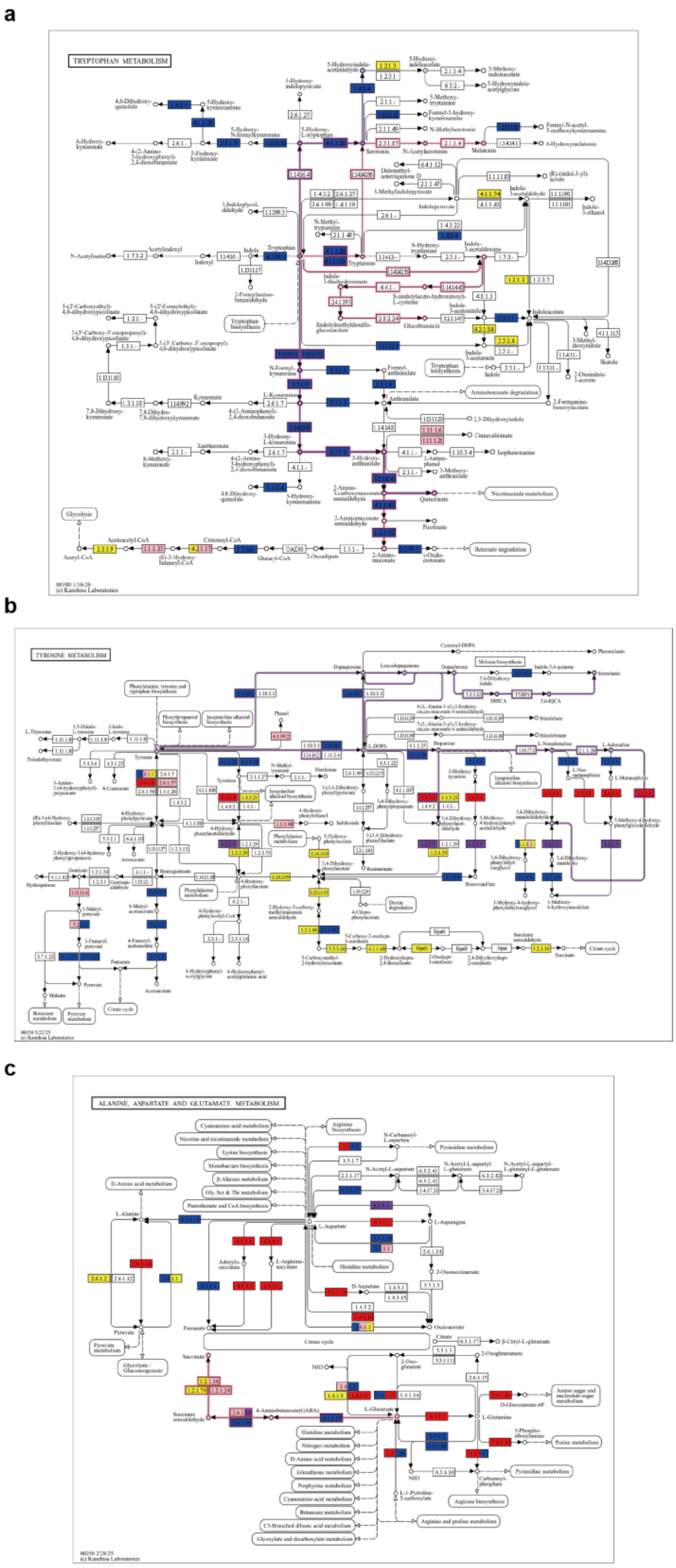

Figure S2 Metabolic Enzyme Abundance Mapping of Amino acid Metabolic Pathways across the Three Cohorts.

(a) Tryptophan Metabolism KEGG Map Diagram; **(b)** Tyrosine Metabolism KEGG Map Diagram; **(c)** Alanine, aspartate and glutamate metabolism KEGG Map Diagram; Colors indicate the abundance level of each group. Yellow: Dep-adolescent > Dep-adult > Con-adolescent; Pink: Dep-adult > Dep-adolescent > Con-adolescent; Blue: Con-adolescent > Dep-adult > Dep-adolescent; Purple: Dep-adolescent > Con-adolescent > Dep-adult; Red: Con-adolescent > Dep-adolescent > Dep-adult; Green: Dep-adult > Con-adolescent > Dep-adolescent.
